# Effects of Whole-Body Cryostimulation on Pain Management and Disease Activity in Active Rheumatic Polymyalgia: A Case-Report

**DOI:** 10.3390/biomedicines11061594

**Published:** 2023-05-31

**Authors:** Federica Verme, Alessandra Scarpa, Giorgia Varallo, Paolo Piterà, Paolo Capodaglio, Jacopo Maria Fontana

**Affiliations:** 1 Research Laboratory in Biomechanics, Rehabilitation and Ergonomics, Istituto Auxologico Italiano, IRCCS, San Giuseppe Hospital, 28824 Piancavallo, Italy; p.pitera@auxologico.it (P.P.); p.capodaglio@auxologico.it (P.C.); j.fontana@auxologico.it (J.M.F.); 2 Psychology Research Laboratory, Istituto Auxologico Italiano, IRCCS, 20145 Milano, Italy; a.scarpa@auxologico.it; 3 Department of Medicine and Surgery, University of Parma, 43121 Parma, Italy; giorgia.varallo@unipr.it; 4 Department of Surgical Sciences, University of Torino, Physical Medicine and Rehabilitation, 10121 Torino, Italy

**Keywords:** polymyalgia rheumatica, whole-body cryostimulation, chronic pain, disease activity, rehabilitation

## Abstract

Polymyalgia rheumatica (PMR) is the most common inflammatory rheumatic disease among people over 50 and occurs with symptoms such as musculoskeletal pain and stiffness in the neck, shoulders, and hips. To date, corticosteroids represent the cornerstone of PMR treatment. However, it is well known that their prolonged use is associated with several adverse effects, making it crucial to find therapeutic alternatives. The purpose of this case report was to describe the effectiveness of 10 whole-body cryostimulation (WBC) sessions on a 74-year-old woman suffering from PMR. An improvement in disease impact, fatigue, pain, quality of sleep, and total physical activity was observed after WBC. Moreover, the patient reduced her daily drug intake by 67% following WBC treatments. Given the increasing prevalence of PMR and considering the side effects that drug treatments can lead to, WBC could represent a valuable adjuvant and well-tolerated alternative for treating PMR.

## 1. Introduction

Polymyalgia rheumatica (PMR) is the most common inflammatory disease in elderly people of North European ancestry. It affects people over the age of 50 (2–3 times more common among women), with an average age of onset just over 70 and a higher prevalence in Scandinavian countries and people of northern European descent. Patients with PMR usually present proximal muscle pain and stiffness of acute or subacute onset in the neck, shoulders, upper arms, hips, and thighs, resulting in a pronounced morning stiffness with difficulties in performing some daily activities, such as turning over in bed, rising from a bed or a chair, or getting dressed [1,2]. Patients often report constitutional symptoms such as asthenia, anorexia, weight loss, and low-grade fever [3,4,5].

Whole-body cryostimulation (WBC) is a physical treatment that exposes the entire body to cryogenic temperatures (−110 °C to −140 °C) for a short period (2–3 min). Exposure to these temperatures can reduce pain and inflammatory status improving several metabolic parameters such as thermogenesis, lipid profile, insulin sensitivity, and glucose utilization [6,7], but also depression, anxiety [8], and sleep quality [9]. Moreover, cycles of WBC are able to reduce fatigue and disease activity in patients with several conditions, such as multiple sclerosis [10], post-COVID-19 condition (PCC) [11,12], rheumatoid arthritis [13], ankylosing spondylitis [14], and fibromyalgia [6,15,16].

The cryostimulation analgesic and anti-inflammatory effect is exerted through the sudden thermal stress which stimulates the cutaneous thermal receptors by lowering skin temperature, slowing down nerve conduction in pain fibers, and causing vasoconstriction and pain modulation via inhibitory action through pathways that carry pain-related signals. Repeated exposure of the body to WBC decreases the production of pro-inflammatory and oxidative substances. These effects add up to an increased parasympathetic tone by decreasing sympathetic nerve activity, resulting in reduced fatigue sensation and muscle tension, delayed muscle soreness, and improved mood and symptoms of depression [6].

It is also important to consider that despite its widespread use worldwide, WBC is associated with relatively infrequent and mostly mild and transient adverse effects [17].

To the best of our knowledge, no studies have so far investigated the effects of WBC on PMR. Given the physiological effects of WBC and the underlying scientific evidence, we exposed a 74-year-old woman diagnosed with PMR to 10 sessions of WBC, to evaluate its effectiveness on symptom management.

## 2. Detailed Case Description

In September 2022, due to the persistence of myalgia, R.A., a 74-year-old Italian woman with a BMI of 20.9 kg/m^2^, diagnosed with PMR, osteoporosis, and psoriasis two years earlier (September 2020), came to our outpatient facility at San Giuseppe Hospital (Istituto Auxologico, Piancavallo, Italy) to undergo 10 sessions of WBC.

She was undergoing the following pharmacological therapy: prednisone 3.75 mg daily, alendronate 70 mg per week, and vitamin D therapy (50,000 UI, monthly).

Because the patient reported pain at the pelvic girdle and the right shoulder and night-morning stiffness, a radiological examination was performed, and no major pathological findings were detected.

The patient underwent four successive follow-ups in which the cortisone dose was adjusted each time by the physician based on the patient’s reported symptoms, until replacement with a steroid-sparing agent (methotrexate) and a gamma–amino–butyric acid analog (pregabalin) and final reintroduction (June 2022) due to their ineffectiveness in relieving persistent widespread pain, stiffness, and myalgia (Table 1).

Before PMR diagnosis and in the four follow-ups thereafter (December 2020, May 2021, October 2021, April 2022), complete blood count (red and white blood cells, hemoglobin, hematocrit, and platelets), chemistry parameters (glucose, cholesterol, and triglycerides) and levels of thyroid stimulating hormone and free thyroxine (FT4) were within normal ranges.

In addition, prior to starting the WBC sessions, the patient reported a moderately active life that consisted, on average, of daily walks and light exercise activities two to three times a week. However, she was experiencing persistent pain and reduced energy.

Whole body cryostimulation. From 19 September to 23 September 2022, R.A. underwent 10 total WBC sessions. The treatments were performed twice a day, the first at 9 a.m. and the second at 12 p.m. inside a cryo-chamber (Artic, CryoScience, Rome) where she was exposed to extremely cold, dry air at −110 °C for two minutes. Before starting the treatment, the patient was medically screened for contraindications according to Bad Voslau’s guidelines [18]. She was asked to remove glasses and metal accessories and to wear minimal clothing, including a T-shirt, running shorts, an earmuff band, gloves, socks (pulled up to the knee), and rubber slippers. Trained personnel supervised the entire procedure and instructed the patient to breathe calmly and slowly throughout.

Primary outcome measures. The patient was asked to complete some questionnaires to assess her physical condition before undergoing WBC (September 2022) and five months after completing the treatments (February 2023). The fibromyalgia impact questionnaire (FIQ), in its Italian validation [19], was used to measure the patient’s disease status, progress, and outcomes. Total scores range from 0 to 100, where higher scores indicate a more severe impact of FM. Fatigue severity scale (FSS) [20] is a 9-item questionnaire used to assess the severity of fatigue symptoms, whose items are scored on a 7-point scale (1 = strongly disagree, 7 = strongly agree), where a higher score corresponds to greater symptom severity. A numeric rating scale (NRS) [21] ranging from 0 to 10 was used to measure pain intensity, where high scores indicated high levels of pain intensity. A pain management questionnaire [22] was administered to assess the medications taken and their dosages. Pittsburgh sleep quality index (PSQI) was used to evaluate sleep quality and disturbances over a 1-month interval. It consists of nineteen individual items which generate seven “component” scores (subjective sleep quality, sleep latency, sleep duration, habitual sleep efficiency, sleep disturbances, use of sleeping medication, and daytime dysfunction), whose sum yields one global score [23]. The International Physical Activity Questionnaire-Short Form (IPAQ-SF) [24] is widely used to assess physical activity. It consists of questions about an individual’s physical activity (PA) over the past 7 days. The IPAQ-SF is classified into three categories: low-intensity activity, which consists of walking of any kind; moderate-intensity activity, which causes breathing to be more difficult than normal levels; and vigorous physical activity, which makes breathing much more difficult than normal. Results can be reported in categories (low activity levels, moderate activity levels, or high activity levels) or as a continuous variable (MET minutes a week). MET minutes represent the amount of energy expended carrying out physical activity. 

Results. These questionnaire scores completed by the patient immediately before undergoing WBC and then 5 months after concluding the treatments were compared (Table 2). FIQ and FSS scores showed a clear improvement in the patient’s disease impact (pre-WBC score = 46.17, post-WBC score = 14.17), and fatigue symptoms (pre-WBC score = 44, post-WBC score = 29). The patient also reported improved pain symptoms (NRSpre-WBC score = 9, post-WBC score = 3), to the extent that she reduced the corticosteroid to 1.25 mg per day. In addition, the patient reported better sleep quality following WBC treatments, supported by PSQI total score going from 7 (pre-WBC) to 0 (post-WBC). Finally, IPAQ shows that the patient was very active before and after WBC (>3000 MET.min/week), but a clear increase in total PA MET.min/week was observed over the 5 months of the study (total IPAQ score pre-WBC = 7110, post-WBC = 19,680). Notably, the patient did not report vigorous activity, but her moderate activity increased from 1440 to 9600 MET.min/week, while her walking score increased from 5670 to 10,080 MET.min/week.

Complete blood count (red and white blood cells, hemoglobin, hematocrit, and platelets) and chemistry (glucose, cholesterol, and triglycerides) performed by the patient four months after WBC treatments (January 2023) were within normal ranges.

## 3. Discussion

The main goals of rehabilitation treatments are to control the disease and prevent relapses, which are quite common. To date, despite the availability of new and costly drugs, corticosteroids (oral prednisone/prednisolone constitutes) remain the cornerstone of treatment and are still unsurpassed in terms of symptom resolution and control of inflammation [3,4].

However, it is well known that prolonged use of corticosteroids can lead to a wide array of adverse effects ranging from mild to severe [25]; therefore, finding therapeutic alternatives to their use becomes crucial.

To date, no studies have investigated WBC as an adjuvant option for treating PMR. Given the known effects of WBC on reducing pain and disease impact in patients with fibromyalgia, and on improving the inflammatory profile in patients with RA [26,27,28], this study aimed at evaluating the effect of WBC on pain severity, disease impact, fatigue, and sleep quality on a patient suffering from PMR. The results show that the patient reduced her daily drug intake by 67% following WBC treatments. Moreover, she reported that the effects lasted over time, still experiencing benefits 5 months after the end of the treatments. These results align with other findings where the effect of WBC was also evaluated at follow-up visits in patients with FM and RA. Specifically, in a study by Vitenet et al. [16], a group of patients with fibromyalgia diagnosis who underwent 10 sessions of whole-body cryostimulation reported improved health-related quality of life even 1 month after treatment discontinuation. In addition, in a recent randomized controlled trial [26], patients with RA who underwent WBC sessions reported lower pain levels compared to baseline, even twelve weeks after the end of the treatments. Interestingly, some of these patients reported at follow-up that they had reduced or discontinued using analgesics.

Improvements in pain, fatigue, disease impact, and quality of life have been reported in other studies [6,13,15,29,30], but the duration of benefits has not been evaluated in follow-ups.

Because chronic pain negatively affects mental health, often being associated with anxiety and depression [31], addressing pain management could also be effective in improving patients’ well-being and quality of life. The study by Varallo et al. [15], in which ten sessions of WBC over two weeks produced positive effects in terms of pain severity, depression, disease impact, and sleep quality, clearly indicates that WBC could help to improve some psychological aspects, as well as suggesting that pain reduction may facilitate adherence to the physical activity intervention with a positive effect on pain management and the person’s emotional well-being. However, despite the promising results we obtained, we are aware that 10 sessions of WBC are insufficient to produce a significant overall improvement. For this reason, future studies with longer WBC interventions will be evaluated.

## 4. Conclusions

PMR is not a rare disease, and its prevalence will continue to increase in the coming years as the number of elderly people worldwide continues to rise. Once diagnosed, further proper management of patients it’s crucial to significantly improve the patient’s prognosis. Therefore, as demonstrated in this report, WBC could be a valuable adjuvant for the treatment of PMR that could potentially have a positive effect in cases of poor prescription compliance and terms of health care costs. Indeed, in this specific case, WBC is a scalable and well-tolerated alternative aimed at reducing not only pain but also fatigue, disease impact, drug therapies, and largely improving sleep and physical activity.

As this is the first work on the effects of WBC in patients with PMR, further research is obviously needed to confirm the data obtained. Since WBC treatment was conducted on a single patient with no comorbidities, no overweight problems, and a moderately active lifestyle, a larger and more diverse sample will therefore be needed for future studies to confirm the adjuvant role of WBC in the treatment of PMR and other conditions characterized by chronic persistent pain. Because of the lack of studies on the response of WBC concerning age and sex, this aspect also deserves further investigation.

WBC should not be considered as a once-in-a-lifetime treatment. In fact, parameters such as exposure temperatures, number of WBC sessions, and long-term duration of WBC effects should be defined according to the clinical characteristics, age, sex, and goals of the subjects to maximize beneficial effects and design a tailored therapeutic and rehabilitation program.

## Figures and Tables

**Table 1:** List of symptoms and medications prescribed to the patient from September 2020 to subsequent follow-ups.

Follow-Up Date	Reported Symptoms	Prescribed Medications
September 2020 (Diagnosis of PMR)	Pain (pelvic girdle and right shoulder), stiffness, psoriasis.	Prednisone: 25 mg daily, for five days; 18.75 mg daily for five days; 12.5 mg daily for 14 days; 10 mg daily until follow-up.
December 2020	Girdles arthralgias.	Prednisone: 10 mg daily for ten days, then gradually reduce up to 5 mg daily until follow-up.
May 2021	Widespread pain, morning stiffness, hands arthralgias.	Prednisone: 7.5 mg daily, for two weeks; 6.25 mg daily, for a month; 5 mg daily for a month; 2.5 mg daily for a month; stop until follow-up.
October 2021	Widespread stiffness and pain, arthralgias, psoriasis.	Methotrexate: 10 mg per week. Prednisone: 5 mg daily for two months; 3.75 mg daily per month.
April 2022	Widespread pain, neck pain.	Pregabalin: 25 mg daily; if well tolerated, increase to 75 mg daily.

**Table 2:** Pre-post WBC10 total scores and percentage change of FIQ (disease impact), FSS (fatigue), NRS (pain), PSQI (sleep), IPAQ (physical activity) questionnaires, and medication dosage (Prednisone). The percentage change (Δ%) was calculated using the formula: Δ% = (POST−PRE)/PRE × 100.

Questionnaires (Total Scores)			
	PRE-WBC_10_	POST-WBC_10_	Δ%
FIQ (disease impact)	46.17	14.17	−69.3
FSS (fatigue)	44	29	−34.1
NRS(pain)	9	3	−66.7
PSQI (sleep)	7	0	−100
IPAQ (physical activity)	7110	19,680	+176.8
Medication (total dosage)			
	PRE-WBC_10_	POST-WBC_10_	Δ%
Corticosteroid	(Prednisone) 3.75 mg	(Prednisone) 1.25 mg	−66.7

## Data Availability

The data presented in this study are available within the article.
